# A Randomized Controlled Trial Evaluating a Manualized TeleCoaching Protocol for Improving Adherence to a Web-Based Intervention for the Treatment of Depression

**DOI:** 10.1371/journal.pone.0070086

**Published:** 2013-08-21

**Authors:** David C. Mohr, Jenna Duffecy, Joyce Ho, Mary Kwasny, Xuan Cai, Michelle Nicole Burns, Mark Begale

**Affiliations:** Center for Behavioral Intervention Technologies, Department of Preventive Medicine, Northwestern University Feinberg School of Medicine, Chicago, Illinois, United States of America; Catholic University of Sacred Heart of Rome, Italy

## Abstract

**Background:**

Web-based interventions for depression that are supported by coaching have generally produced larger effect-sizes, relative to standalone web-based interventions. This is likely due to the effect of coaching on adherence. We evaluated the efficacy of a manualized telephone coaching intervention (TeleCoach) aimed at improving adherence to a web-based intervention (moodManager), as well as the relationship between adherence and depressive symptom outcomes.

**Methods:**

101 patients with MDD, recruited from primary care, were randomized to 12 weeks moodManager+TeleCoach, 12 weeks of self-directed moodManager, or 6 weeks of a waitlist control (WLC). Depressive symptom severity was measured using the PHQ-9.

**Results:**

TeleCoach+moodManager, compared to self-directed moodManager, resulted in significantly greater numbers of login days (*p* = 0.01), greater time until last use (*p* = 0.007), greater use of lessons (*p* = 0.03), greater variety of interactive tools used (*p* = 0.02), but total instances of tool use did not reach statistical significance. (*p* = 0.07). TeleCoach+moodManager produced significantly lower PHQ-9 scores relative to WLC at week 6 (*p* = 0.04), but there were no other significant differences in PHQ-9 scores at weeks 6 or 12 (*p*s>0.20) across treatment arms. Baseline PHQ-9 scores were no significantly related to adherence to moodManager.

**Conclusions:**

TeleCoach produced significantly greater adherence to moodManager, relative to self-directed moodManager. TeleCoached moodManager produced greater reductions in depressive symptoms relative to WLC, however, there were no statistically significant differences relative to self-directed moodManager. While greater use was associated with better outcomes, most users in both TeleCoach and self-directed moodManager had dropped out of treatment by week 12. Even with telephone coaching, adherence to web-based interventions for depression remains a challenge. Methods of improving coaching models are discussed.

**Trial Registration:**

Clinicaltrials.gov NCT00719979

## Introduction

The one-year prevalence rate of major depressive disorder (MDD) has been estimated between 6.6–10.3% in the general population [1], [2], taking an enormous toll in terms of cost, morbidity, suffering, and mortality [3]–[5]. While most depressed people want psychological care as part of their treatment [6], most also experience substantial access barriers that prevent care [7], [8].

Web based interventions have increasingly been investigated as low intensity treatments for depression that potentially have greater reach than more intensive psychotherapy. Trials of standalone web-based treatments have shown very weak effects, while treatments involving coach support have produced stronger results [9], [10]. The weaker effects are likely due to the very high dropout rates and non-adherence seen in standalone treatments [11], [12]. Coached interventions typically involve less than 2 hours of the coach's time, and are commonly delivered via email, secure messaging, or telephone. While trials of coached interventions have produced better adherence, dropout rates for web-based interventions remain higher for MDD than for other disorders [13].

A large body of research would suggest that coaching is a critical component of web-based interventions, yet very little work has been done to understand, define, and evaluate coaching. Coaching components of web-based intervention trials are typically not manualized and usually not defined in trial reports. Manualization is an important step in intervention development, as it documents the intervention steps tested and supports reliable dissemination. Manualization also supports the refinement of procedures, through continuing, iterative development.

We have described a model of coaching, called “supportive accountability [14],” for behavioral intervention technologies (BITs), including web-based and mobile interventions. Supportive accountability focuses solely on improving adherence to interventions, and not on the clinical intervention components, which are provided by the web-based intervention. Supportive accountability posits that adherence to BITs can be improved through accountability to a coach who is seen as trustworthy, benevolent, and having expertise. Use goals should involve clear, process-oriented expectations that the patient is involved in determining. Accountability involves expectations that the patient will communicate with the coach about the achievement or failure to achieve use goals. Reciprocity in the relationship, through which the patient derives clear benefits, should be explicit. A strong therapeutic bond is expected to enhance the effects of accountability.

We developed a coaching manual, TeleCoach [15], based on supportive accountability, in which defined coach support is delivered via telephone. An 8-week single arm pilot study of TeleCoach support for our web-based depression intervention, moodManager, found a dropout rate under 10% and a very large reduction in depression [16]. The TeleCoach model is distinct from many other coaching models in that it focuses entirely on promoting adherence. Coaches are not permitted to provide treatment or counseling for life problems or to guide the clinical aspects of site use, such as describing how to use an activity scheduler or thought record. The focus on adherence is intended to more cleanly separate treatment functions, which were provided by the website, and site use motivation, which is supported by the coach. In addition, the TeleCoach protocol was designed to support scalability in that it does not require mental health expertise to administer.

The aim of this study was to pilot the utility of TeleCoach support for moodManager, relative to a standalone deployment of moodManager and wait-list control (WLC). It was hypothesized that TeleCoach would improve adherence to moodManager relative to standalone moodManager. Further, it was hypothesized that moodManger with TeleCoaching would produce greater reductions in depressive symptoms, compared to a wait-list control and to moodManager alone. The relationship between adherence to moodManager and depressive symptom severity was explored.

## Methods

### Participants

Participants were recruited from July 2009 to February 2011 through the Northwestern University General Internal Medicine clinic via fliers in exam rooms, physician referral, and recruitment letters sent by postal mail to randomly selected clinic patients (selection based on mental health information is prohibited by law in Illinois). Participants were also recruited through physician referral and fliers from General Internal Medicine clinics that were part of the REACH practice-based research network affiliated with the Northwestern University Clinical and Translational Sciences Institute. Inclusion criteria consisted of diagnosis of current Major Depressive Disorder (MDD) on the telephone administered Mini-International Neuropsychiatric Interview (MINI) [17], a score of 11 or greater on the telephone administered Quick Inventory of Depressive Symptomatology (QIDS) [18], age 18 or older, ability to speak and read English, and access to a telephone and computer with broadband access to the internet. People were excluded if they had a severe psychiatric disorder (e.g. psychotic disorder, dissociative disorder, etc.) based the MINI, reported substance or alcohol abuse that would interfere with treatment, met criteria for dementia on the Telephone Interview for Cognitive Status [19], were currently receiving or planning to receive psychotherapy, had initiated pharmacotherapy for depression in the previous 10 days, or reported severe suicidality including a plan and intent.

### Study Procedures

The Northwestern University Institutional Review Board approved study procedures. Interested individuals could contact study staff via telephone or email. Interested individuals received a brief depression screen to assess preliminary eligibility. Those who evidenced signs of depression were consented verbally over the telephone and were mailed or emailed the consent document, which they signed and returned. Following the verbal consent, interested individuals received a telephone interview and were provided questionnaires via a secure online survey. The protocol for this trial and supporting CONSORT checklist are available as supporting information; see Checklist S1 and Protocol S1.

Participants were randomly assigned by a statistician to receive 12 weeks of our web-based treatment for depression, moodManager with weekly telephone coaching, 12 weeks of self-directed, standalone moodManager, or a 6-week wait-list control (WLC). Computer generated randomization was conducted on a 1∶1∶1 ratio in blocks of 6, stratified by pharmacotherapy status. The statistician was blinded to baseline assessment to prevent allocation bias. Although all participants were under the care of a primary care physician, the WLC was restricted to 6 weeks, as it was determined to be unethical to ask participants with MDD to wait a full 12 weeks. Participants were assessed by telephone interview and online questionnaires at baseline, week 6 (WLC endpoint), Week 12 (end of moodManager interventions), and Week 16 (4 week post-treatment follow-up). To minimize loss-to-follow-up, participants were paid up to $100 for completion of assessments. Participants were clearly informed that payment was not for use of the website, to ensure that payments did not influence treatment adherence. Study staff continued to contact participants who had discontinued treatment to obtain outcome assessments.

### Treatments

***moodManager*** provided participants with a secure, password-protected access to an expanded version of a web-based cognitive behavioral therapy (CBT) program that has been previously evaluated [16].

This version of moodManager included 18 lessons, each with accompanying tools. Lessons included text, graphics, audio and video, and were intended to teach basic CBT concepts, and demonstrate to participants how to use the tools. Lessons require 10–20 minutes to complete. Tools were designed to support implementation of cognitive behavioral skills, required only 1–3 minutes to complete, and were intended to be used 3–4 times per week. There were 5 different types of tool templates, which contained scaffolded instantiations to match each lesson. For example, the Activity Diary had several iterations, including one that was solely for activity monitoring, one that provided activity scheduling, and one that combined both scheduling and monitoring. The Thought Diary tool included a thought monitoring version, a version that included challenging unhelpful thoughts, and one that supported the identification of thought distortions. The Action Experiment tool combined the Activity Diary tool and the Thought Diary tool in order to help people practice activities based on a restructured cognitive interpretation of an event. moodManager also contained mood rating and tracking features for self-monitoring by patients.

The first 7 lessons and tools provided basic training in CBT skills and include the following: 1) “Getting Started” is an introduction to the basic principles of CBT; 2) “Monitoring Activities,” describes the relationship between activities and mood, and introduces the Activity Diary tool that allowed participants to track and rate daily activities; 3) “Scheduling Positive Activities” teaches participants to use the Activity Scheduler to plan and schedule positive activities; 4) “Identifying Thoughts” describes the effects of thoughts on mood and teaches participants to use the Thought Diary tool to monitor automatic thoughts; 5) “Thought Distortions” describes common cognitive distortions and extends the thought tool to label unhelpful thought patterns; 6) “Challenging Thoughts” expands the Thought Diary tool by teaching participants to develop alternative thoughts; 7) “Action Experiments” links activities and thoughts, allowing participants to schedule behavioral experiments to test their unhelpful thoughts. Progression through these core modules was based on time and completion rules. Participants were required to complete a tool three times before the next learning module would open, to ensure that the concept was learned. However, to allow patients to progress, patients who did not use the tool three times were moved ahead after one week. Coaches could also “bump” patients ahead to the next lesson.

The remaining 11 lessons and tools are opened upon completion of the core modules and are designed to address common problems and comorbidities associated with depression. Users can select any lesson/tool set, or can complete a brief questionnaire for guidance. Lesson/tool sets include Anxiety and Worry, Relaxation, Mindfulness, Guilt/Shame, Anger Management, Improving Personal Relationships, Communication Skills Assertiveness, Coping, Living Your Strengths and Values, and Maintaining Gains.

The moodManager site also contains a coach interface that allows the coach to observe the patient's activity on the site, including dates and times of site visits, content of patient's entries into tools (e.g. activity diaries and thought records), depression monitoring ratings, and alerts for suicidality.

***TeleCoaching*** involved weekly 5–10 minute calls focused on maintaining adherence to moodManager. The TeleCoach protocol was based on principals of Supportive Accountability [14]. TeleCoaching focused on enhancing adherence to moodManager by establishing a supportive relationship, setting and reviewing login goals, positively reinforcing login and site use, encouraging use of moodManager when login goals were not met, and answering any questions regarding the functionality of the site. Discussions related specifically the CBT content of moodManager were not permitted. When participants raised questions about life difficulties, the coach could suggest areas of the website to explore, but did not engage in a discussion of the life problem with the patient and did not assist the patient in the therapeutic use of the site. Restriction of the TeleCoaching protocol to adherence as intended both to make the protocol capable of being administered by non-mental health clinicians, and to evaluate the capacity of the website to administer all treatment components of the intervention. The TeleCoach protocol was operationalized in a treatment manual [15] that has been field-tested [16].

The coaches were two masters level social workers and two Ph.D. level psychologists. Coaches spoke weekly by telephone with participants and were available by email. Participants received an initial “engagement session,” lasting 30–45 minutes, to establish a bond, convey benevolence and expertise, and discuss treatment expectations. Subsequent conversations with the coach were intended to be 5–10 minutes in length. Participants were also permitted to email their coaches with questions during the week.

All coaching sessions were audiotaped. Coaches received an initial half-day training and group supervision, which was initially weekly, dropping in frequency as mastery was established

***Wait-List Control*** participants were not provided any intervention for 6 weeks, after which they were allowed to choose coached or self-directed moodManager. The WLC condition was intended to verify that the coached and self-directed conditions had an effect on depressive symptoms. As in all conditions, participants in the WLC received other forms of care from their primary care physician, which generally include consultation and pharmacotherapy.

### Assessment

#### Personal Health Questionnaire (PHQ-9)

The PHQ-9 [20] was the primary outcome measure. This is a 9-item self-report measure of depressive symptoms that closely matches the DSM-IV criteria for major depressive episode (MDE) and was administered online at all time points.

#### Mini International Neuropsychiatric Interview (MINI)

The MINI [17] is a structured interview to evaluate DSM-IV Axis I disorders, including Major Depressive Episode (MDE), as well as most other common diagnoses. The full MINI was administered by telephone at screening, but only the MDE section was administered at follow-up telephone evaluations. The telephone interviews were administered and audiotaped by trained clinical evaluators who were blind to the coaching component of the study, site usage and outcome data. Evaluators received extensive training and supervision by a PhD level psychologist (JH) on assessment protocols.

#### Telephone Interview for Cognitive Status (TICS)

This is a widely used telephone assessment that has demonstrated reliability and validity in identifying dementia resulting from Alzheimer's and stroke [19]. The TICS was administered to screen for dementia prior to enrollment.

#### Adherence to moodManager

Patient adherence to moodManager was measured by logins. To prevent artifacts, such as high logins due to connectivity problems, number of days logged in during the study period was used as the primary adherence measure. To evaluate length of engagement, we examined number of days until last login. The intervention components were investigated by examining number of times lessons were viewed, the number of times tools were used, and the number of different types of tools used. Because multiple tools could be used in a single session, all instances of tool use were included in the number of tool uses.

#### Participant Adherence to TeleCoach

Number of completed coaching sessions and weeks to final TeleCoaching sessions were used as markers of participant engagement. Length of the TeleCoach sessions was also determined by the length of the audiotaped session.

#### Adverse Events

Suicidality, hospitalizations and other adverse events were monitored through telephone evaluations at each assessment point, and through coach supervision.

### Statistical Analyses

Baseline demographic and depression variables were compared across the three treatment groups using Chi-square tests and one-way ANOVA. Rates of lost-to-follow-up were compared between moodManager and TeleCoaching groups using chi-squared tests. Data on Coach adherence is presented as mean and standard deviation. Adherence levels to moodManager were skewed, and are therefore presented by group with the median, 25^th^ and 75^th^ percentiles, minimum and maximum, and compared using Wilcoxon Rank Sum tests. Additionally, adherence was modeled using Poisson regression with the total number of possible access days as an offset, adjusting for group to determine if baseline depression or demographics affected adherence levels.

Depression outcomes were analyzed on an intention-to-treat basis. Mixed model analyses were used to compare depression outcomes over time in TeleCoached moodManager, relative to self-directed moodManager and WLC at 6 weeks, given that the WLC ended at that point, and between TeleCoached and self-directed moodManager at 12 weeks. While mixed models can account for missing data [21], these approaches require three time points. Accordingly, multiple imputation was used, in which 20 datasets were created, imputing values for missing data based on the observed data [22]. Test results across the imputed datasets were combined using multiple imputation combining rules described by Li [23]. Depression outcomes are presented as least square means and standard errors, and effect sizes are presented as least squares mean differences. Spearman's correlation, linear and logistic regression models were also fit to examine the effects of adherence on depression outcome.

As this was a pilot trial, the aim was to establish feasibility for a larger trial. Sample size was not based on power. Based on the sample size, and power of .80, the current trial was powered to detect standardized effect sizes between any two groups of 0.66 using independent t-tests, within groups of 0.50, and correlations of 0.33 or higher.

## Results

**Figure 1:**
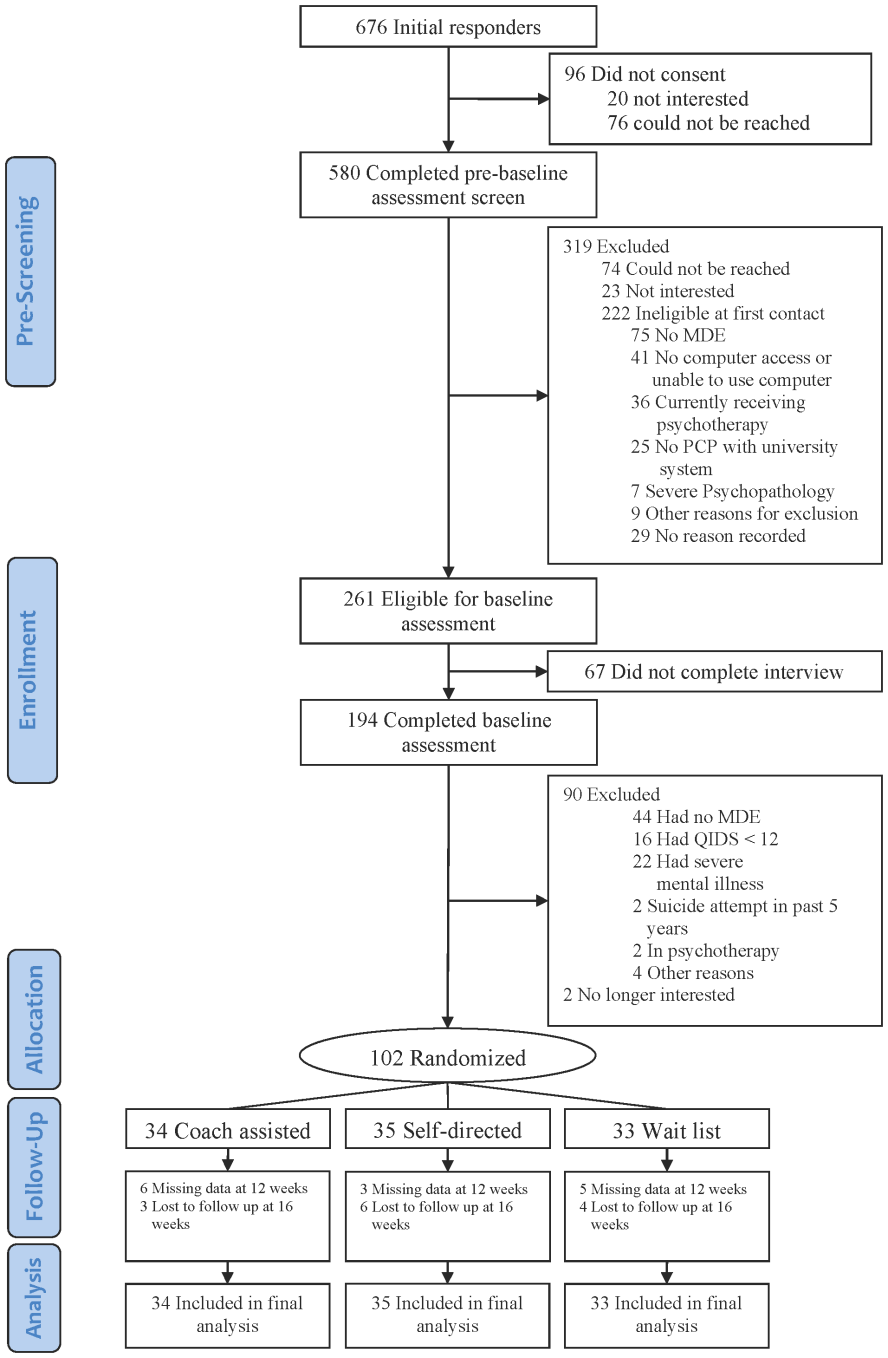
Flow of study participants through the trial.

The flow of participants is shown in Figure 1. There were no significant differences in the lost-to-follow-up rate across treatment arms; the lost-to-follow up rate was 29.4% in Coach group, and 17.1% in Self-Directed group (*p* = 0.36). Table 1 summarizes the baseline demographics and characteristics of the participants. There were no significant differences in these baseline variables across treatment arms.

**Table 1:** Baseline Participant Characteristics.

Variable	Total N	Coach			Self Directed			WLC			P-value
		N	Mean	SD	N	Mean	SD	N	Mean	SD	
**Age**	**102**	34	47.6	12.4	35	48.9	14.6	33	48.49	11.7	0.91
PHQ9 total score	**102**	34	15.7	5.2	35	15.5	4.3	33	15.4	5.2	0.96
	**N/%**	**N**	**%**		**N**	**%**		**N**	**%**		
**Gender**	**102**										0.94
Female	73 (71.4%)	25	73.5%		25	71.4%		23	69.7%		
**Ethnicity** *	**98**										0.84
Not Hispanic or Latino	91 (92.9%)	30	93.8%		32	94.1%		29	90.6%		
**Race** **	**101**										0.64
African American	34(33.7%)	14	41.2%		9	26.5%		11	33.3%		
White	60(59.4%)	19	55.9%		22	64.7%		19	57.6%		
Other	7(6.9%)	1	2.9%		3	8.8%		3	9.1%		
**Education**	**102**										0.85
High school	3(2.9%)	1	2.9%		0	0.0%		2	6.1%		
Some college	25(24.5%)	9	26.5%		9	25.7%		7	21.2%		
Bachelor's Degree	35(34.3%)	13	38.2%		12	34.3%		10	30.3%		
Advanced Degree	39(38.2%)	11	32.4%		14	40.0%		14	42.4%		
**Employment** ***	**94**										0.08
Employed	53(56.4%)	13	40.6%		19	57.6%		21	72.4%		
Unemployed	20(21.3%)	11	34.4%		7	21.2%		2	6.9%		
Disability or Retired	21(22.3%)	8	25.0%		7	21.2%		6	20.7%		
**Marital Status** ****	**100**										0.79
Single	34(34%)	13	38.2%		11	32.4%		10	31.3%		
Married or Living with significant other	48(48%)	17	50.0%		15	44.1%		16	50.0%		
Divorced or widowed	18(18%)	4	11.8%		8	23.5%		6	18.8%		
**Antidepressant Status**	**102**										
	35(34.3%)	12	35.3%		12	34.3%		11	33.3%		0.99
**Psychiatric Comorbidities**	**102**										
Agoraphobia without Panic	10 (9.8%)	2	5.9%		4	11.4%		4	12.1%		0.70
Panic with Agoraphobia	5(4.9%)	4	11.8%		0	0.0%		1	3.0%		0.06
Panic without Agoraphobia	4(3.9%)	2	5.9%		1	2.9%		1	3.0%		0.84
Post traumatic	6(5.9%)	0	0.0%		2	5.7%		4	12.1%		0.08
Social Phobia	19(18.6%)	7	20.6%		5	14.3%		7	21.2%		0.72
Generalized Anxiety Disorder	72(70.6%)	23	67.7%		23	65.7%		26	78.8%		0.45
Obsessive Compulsive Disorder	9(8.8%)	5	14.7%		2	5.7%		2	6.0%		0.46

* Ethnicity had 4 cases not replied.

** Race had 1 case not replied.

*** Employment had 8 cases not replied.

**** Marital status had 2 cases not replied.

### Participant Adherence to moodManager

Adherence data to moodManager over the 12-week trial period is provided in Table 2. Coached moodManager produced significantly more login days (Median = 13; range 0–100), compared to self-directed moodManager (Median = 6; range 1–24; *p* = 0.01). Coached participants continued to use moodManager for a median of 61.5 days (8.8 weeks; range 0–87) compared to self-directed patients, who used it for a median of 38 days (5.4 weeks; range 0–81; *p* = 0.007). Coached moodManager participants viewed a median of 8.5 lessons (range 0–18) while self-directed patients viewed a median of 5 lessons (range 1–18; *p* = 0.03). Coached participants also used a wider variety of tools (median = 3, range 0–5), compared to self-directed participants (median = 2; range 0–5; *p* = 0.02). While participants used tools frequently, the frequency of use only reached trend levels of significance, with coached participants using tools a median of 159 times (range 0–721), compared to a median of 39 for self directed participants (range 0–332; *p* = 0.08).

**Table 2:** Intervention Adherence.

Variable	Group	N	Mean	Median	25th %ile	75th %ile	Minimum	Maximum	*Wilcoxon Rank Sum*	
									*Effect Size*	*p*
Number of days logged in	Coached	34	15.3	13	4	18	0	100	0.30	0.01
	Self Directed	35	7.7	6	2	13	1	24		
Total days to from first to last login	Coached	34	51.4	61.5	27	78	0	87	0.30	0.007
	Self Directed	35	33.3	38	0	64	0	81		
Total number of lessons viewed	Coached	34	9.4	8.5	4	17	0	18	0.26	0.03
	Self Directed	35	5.6	5	2	8	1	18		
Total instances of tool use	Coached	34	168.1	159	3	253	0	721	0.21	0.07
	Self Directed	35	66.1	39	4	70	0	332		
Total types of tools used	Coached	34	2.8	3	1	4	0	5	0.28	0.02
	Self Directed	35	1.9	2	1	3	0	5		

### Participant Adherence to TeleCoach

The mean engagement session time was 43.6 min, SD = 14.7, while the mean time for follow-up sessions was 8.9 (SD = 4.5) minutes. Participants completed a mean of 8.5 (SD = 3.7) TeleCoaching sessions and sent a mean of 9.5 emails (SD = 8.6).

### Depression Outcomes

At Week 6, TeleCoached participants had significantly lower PHQ-9 scores compared to WLC [least squares mean difference −2.98 (−5.86, −0.09); *p* = 0.04]. The difference between TeleCoached and Self directed did not reach statistical significance [least squared mean difference −1.09 (−4.04, 1.86), *p* = 0.47)]. Additionally, there was no statistical difference between TeleCoached and self-directed groups at Week 12 [unadjusted difference = 0.40 (−0.16, 0.95), *p* = 0.16; mixed model comparing trajectories of PHQ-9, *p* = 0.67] or at Week 16 (4-week post-treatment follow-up; unadjusted difference = −1.70 (−4.31, 0.91), *p* = 0.20). The least squared estimated means and standard errors for the PHQ-9 are displayed in Table 3.

**Table 3:** Least square means and standard errors for PHQ-9 depression symptom severity scores.

Group	Baseline	Week 6	Week 12	Within Group *p* at 12 Weeks	Week16 (4 Week Post Tx Follow-Up)
**Coached**	15.71 (0.82)	9.84 (0.90)	7.92 (0.94)	<.0001	5.52(0.89)
**Self directed**	15.51 (0.81)	10.41(0.93)	7.84 (0.85)	<.0001	6.69(0.88)
**Waitlist Control**	15.36 (0.83)	12.51(0.86)	N/A	0.06	N/A

There was no significant difference in MDD diagnosis across treatment arms at 6 weeks (*p* = 0.08), when 10 (34.5%) of coached, 8 (29.6%) self-directed and 18 (56.3%) WLC patients met MDD criteria. Similarly, there was no significant difference in MDD status between coached (6, 24.0%) and self-directed patients (6, 18.8%) at week 12 (*p* = 0.63). There was no significant difference at the Week 16 (4-week post-treatment) follow up (coached, 7.4%, self-directed 15.4%, *p* = 0.42).

### Relationship between Adherence and Depressive Symptom Severity

Baseline PHQ-9 was not significantly related to number of login days (*r_s_* = −0.07, *p* = 0.58), number of days from first to last login (*r_s_* = −0.02, *p* = 0.87), number of lessons viewed (r_s_ = 0.08, *p* = 0.55), variety of tools used (*r_s_* = 0.05, *p* = 0.67), or frequency of tools used (*r_s_* = −0.00, *p* = 0.97).

Improvement on the PHQ-9 at week 12, controlling for baseline PHQ-9 and group allocation, as adherence was related to group, was significantly related to number of login days (*β* = 0.14; 95%CI = 0.03,0.24; *p* = 0.02), number of lessons viewed (*β* = 0.40; 95%CI = 0.12,0.68; *p* = 0.01), total tool usage (*β* = 0.01, 95%CI = 0.003,0.02; *p* = 0.01), and the variety of tools used (*β* = 1.21; 95%CI = 0.22,2.20; *p* = 0.03). Improvement in PHQ-9 was not related to the number of days from first to last login (*β* = 0.04; 95%CI = −0.02,0.09; *p* = 0.35).

### Adverse Events

No adverse events were found.

## Discussion

### Adherence

The TeleCoaching model, based on supportive accountability [14], produced significantly greater adherence to moodManager across most markers, including number of days logged in, time to discontinuation of website use, number of lessons viewed, and types of interactive tools used; the effect number of tools used did not reach significance. This stands in contrast to two recent trials from Farrer [24] and Berger [25] comparing web-based interventions for depression guided by coaching with standalone web-based intervention, which did not find any increase in adherence produced by coaching.

The Farrer trial provided weekly telephone coaching, but reported median usage of only 2 lessons in the coached intervention and 1.5 in the standalone intervention [24], compared to 8.5 lessons and 5 lessons respectively in the current study. There are many differences between the two trials. Farrer used Moodgym, which is a well-validated intervention [26], [27], and recruited from a national telephone helpline, while we used moodManager [16] and recruited from primary care. It may be that patients calling into a helpline, not expecting sustained treatment, were less motivated than the primary care patients in this study, which resulted in basement effects. However, it is also possible that the use of a manualized coaching protocol based on supportive accountability [14] provides greater guidance to coaches with respect to actions required to enhance adherence.

The Berger trial reported mean use of 8.5 lessons using an email coaching protocol, compared to 6.8 lessons in the standalone intervention [25]. While in the Berger trial the difference between these two usage rates was not statistically significant, these rates are closer to the usage rates seen in our trial. Berger's coaching protocol focused on patient use of the site, which was similar to the aims of TeleCoach. Berger recruited through public advertising, which may have recruited a more motivated sample, as participants had to have the initiative to contact the study.

In contrast to the Berger and Farrer trials, our trial demonstrates that coaching using a manualized supportive accountability approach delivered via telephone can improve adherence. This is consistent with qualitative studies indicating that participants in web-based depression interventions want human support to help with discipline in adhering to treatment (accountability), personal contact (bond), and feedback that they are using program correctly [28].

### Depressive Symptom Outcomes

TeleCoached moodManager produced significant improvement in depressive symptom severity, relative to WLC at week 6. However, consistent with Berger and Farrer trials, there was no significant difference between TeleCoached moodManager and self-directed moodManager at any timepoint. These findings stand in contrast to meta-analyses and reviews that consistently find that coached web-based treatments produce larger effect-sizes than uncoached interventions [9], [29].

Part of the discrepancy between the null findings regarding reduction in depressive symptoms between coached and uncoached conditions in trials, and the consistent findings of differences reported in meta-analyses and reviews, may be that the trials have been small and underpowered. The present trial, along with Berger [25] and Farrer [24] all show fairly consistent effects. In addition it is also notable that the coaching conditions in all three of these trials did not include clinical counseling, while many trials examining coached web-based interactions do include clinical coaching [10], [30]. It is also possible that the meta-analyses and reviews, which do not include studies that actually compare coached and uncoached conditions within a trial, are wrong, and the coaching does not provide clinical benefit. However, the absence of significance is not necessarily evidence of absence of effect; given the small sample sizes, it is difficult to draw conclusions from these null outcomes with respect to the ultimate utility of coaching in improving depressive symptoms. Taken together, the findings suggest that continued work is needed to improve coaching, and understanding the relationship between coaching and symptom change.

### Relationship between Adherence and Depressive Symptoms

Four markers of adherence were strongly associated with improvement in depression, including total days logged in, number of lessons used, variety of tools used and number of tools used, even after controlling for baseline depression and group. The last marker of adherence, days from first to last login, was only marginally related, although the variability seen in this measure was much larger than was observed in the other measures. Importantly, baseline depression was not significantly related to adherence, suggesting that the relationship between adherence and symptom severity is unidirectional. The finding that adherence is associated with improvement is consistent with findings that improving adherence to behavioral intervention technologies generally, and web-based interventions in particular, can improve outcomes [31], [32]. Depressed patients, who drop out of web-based interventions, continue to improve as long as they are using the intervention [33], which suggests that keeping them adherent would improve outcomes. Thus, efforts to develop cost-effective, scalable methods of improving adherence are important to maximizing the potential for web-based interventions to reduce depressive symptoms. Yet, while the TeleCoaching protocol did increase adherence to the moodManager website, these increases did not translate into significant improved outcomes.

There are two potential general reasons for the discrepancy between findings that TeleCoach improved adherence, but not outcomes in depression severity. The first is that the increases in adherence were not optimal. The median length of adherence in the TeleCoached condition was 9 weeks, still shy of the 12 weeks of expected treatment. Most trials of web-based interventions for depression are 6–8 weeks in duration [12], perhaps due to difficulties sustaining engagement over longer periods of time. But most face-to-face psychological treatments are much longer [34] and it seems unlikely that web-based interventions will require shorter durations to produce improvement in symptoms. Thus, even the TeleCoach intervention tested in this trial appears to be inadequate at producing the level and length of engagement required for optimal results. The second reason may be that coaching focused on adherence alone, excluding any clinical focus, may not be sufficient for optimal clinical improvement.

Based on our experience in this trial, we believe several improvements to the TeleCoach model are worth investigating.

### Potential Improvement to TeleCoach

#### Provide Support for both Adherence and Clinical Change

The TeleCoaching protocol focused on adherence exclusively both to promote scalability by not requiring mental health expertise and to focus all clinical intervention on the website. These results, suggest that focusing solely on adherence does boost adherence, but this does not necessarily transfer to learning clinically relevant concepts and skills. In essence, we got what we taught. Coaching may need to include greater attention to assisting patients in understanding and implementing therapeutic concepts than is currently part of the TeleCoach manual.

The optimal amount of coach support for clinical aspects of treatment is not clear. In a qualitative study, Gerhards [28] noted that patients want not only support with website adherence and social support, which are features of supportive accountability, but they also want feedback on whether they are on the “right track” in their web-based intervention. This would imply that a relatively light approach of monitoring patient activity, providing positive reinforcement when engagement with online tools is adequate, and offering guidance only when interactions with the website suggest a lack of understanding may be sufficient. At the other end of the spectrum, websites have been used as an adjunct to augment standard psychotherapy [35]. Some focus on the clinical aspects web-based treatments may be administered successfully by coaches without mental health training [36].

#### Delivery Medium

In addition, greater consideration of the coaching medium may be required. To date, coaching has been delivered via email [29], [30], secure messaging [13], telephone [24], or face-to-face [35]. Different media are characterized by different bandwidths for cues; face-to-face provides the full complement of verbal and non-verbal cues, the telephone eliminates visual cues, instant messaging eliminates voice quality, email reduces cues related to response latency, and so on. We selected the telephone, assuming that greater bandwidth creates greater bond, but wanting to preserve the ability to deliver support remotely. However, a number of patients commented that the telephone calls were at times difficult to schedule or awkward, as many patients experienced significant time constraints [7], [8] or had privacy issues when engaging in the coaching calls. In addition, the computer mediated communication literature has revealed that assumptions regarding the superiority of communication media that provide greater cue bandwidth (e.g. phone or video conferencing compared to email or instant messaging) are not supported. In the absence of cues, people tend to make positive inferences, which can enhance relational qualities [14], [37]. That is, leaner communication media can create better rapport. However, when there is a breach in the relationship, the absence of cues may lead to overly negative attributions. Thus, coaching may be enhanced by using multiple communications media, sequencing them based on the wishes of the patient and the requirements for maintaining a productive and supportive relationship.

#### Contact Timing

A third feature of coaching protocols that may be improved is timing. Most protocols have required regular, typically weekly contact, and contact prior to initiation of web-based treatment may further enhance outcomes [38]. The structure of weekly contact is likely a holdover from face-to-face psychotherapy. However, patients' motivation to engage in web-based interventions can vary substantially. Some patients, who are intrinsically motivated [39], may require less coaching support, while others who are more extrinsically motivation may require more support. A focus of coaching may be to shift motivation for web-site use and behavior change from extrinsic to intrinsic. Thus, coach support frequency that matches the needs and motivational status of the patient may be more effective.

### Limitations

This study has several limitations that must be acknowledged. First, the decision to end the WLC after week 6, while taken due to ethical considerations, was scientifically unfortunate in that we cannot determine the efficacy of the moodManager arms relative to WLC at week 12. Second, the sample size is small, which limits power. Third, patients were recruited from primary care shortly after an office visit. Approximately one third of participants were on antidepressants, and some initiated treatment shortly before entering the trial. Randomization should have removed the between treatment comparison effects, however, we cannot rule out the possibility that some of the within treatment effects may have been due to medications. Indeed, the WLC condition did show a marked, albeit non-significant reduction in symptoms. Fourth, because the TeleCoaching protocol was tested in its entirety, we cannot make inferences about the utility of the specific features of the protocol. Thus, we cannot be certain that the supportive accountability components contributed to adherence. Indeed, as we have described, this trial suggests that as currently conceptualized, the model can be improved. Finally, as with many trials, rigorous trial methodology was employed, including screening evaluation and procedures to support the acquisition of follow-up data. A growing literature indicates that such research procedures can substantially improve adherence to web-based interventions [28], [40]. While these procedures were applied consistently across treatment arms, they likely select for participants who are more likely to adhere, support adherence through research contact, and therefore may limit the generalizability of these findings to clinical settings.

### Conclusion

This is the first randomized controlled trial of a manualized coaching protocol aimed at enhancing patient adherence to a web-based intervention for depression. The trial supports the utility of the TeleCoach protocol in enhancing patient adherence to the moodManager intervention for depression, However, while we found that greater adherence was associated with greater reductions in depressive symptoms, we did not find that depressive symptom severity in the TeleCoached treatment was any better than in the self-directed condition. The use of the manual serves to define the coaching intervention at this early stage. A treatment manual allows for the protocol to be challenged, thereby supporting iterative development and refinement. These data, in conjunction with the larger literature, suggest a number of potential avenues for further improvements in coaching models, including incorporating a greater therapeutic focus, more thoughtful use of multiple communications media, and reconsidering the timing of contact.

## Supporting Information

Protocol S1 **Study Protocol.**
(DOC)

Checklist S1 **CONSORT Checklist.**
(DOC)
