# Supplementary material for: A Randomized Controlled Trial Evaluating a Manualized TeleCoaching Protocol for Improving Adherence to a Web-Based Intervention for the Treatment of Depression
Source: PLoS One. 2013 Aug 21;8(8):e70086. doi: 10.1371/journal.pone.0070086 (PMC3749146; doi:10.1371/journal.pone.0070086)
Supplement: Protocol S1 — Study Protocol. (DOC) [file pone.0070086.s001.doc]

PROTOCOL SUBMISSION TO

NORTHWESTERN UNIVERSITY

INSTITUTIONAL REVIEW BOARD

09/27/10

INTEGRATED TELEMENTAL HEALTH INTERVENTION FOR DEPRESSION

Principal Investigator:

David C. Mohr, Ph.D.

Department of Preventive Medicine, Feinberg School of Medicine

**2. BACKGROUND**

Depression is common, with one-year prevalence rates of major depressive disorder (MDD) estimated to be between 6.6-10.3% in the general population ^1, 2^. Depression imposes a very high societal burden in terms of cost, morbidity, suffering, and mortality ^3-5^. Research has consistently found psychotherapy and pharmacotherapy to be equivalent in efficacy ^6-9^, however, primary care patients have consistently been shown to prefer psychotherapy or counseling to pharmacotherapy ^10-14^. Yet only about 20% of all patients referred for psychotherapy ever enter treatment ^10, 15^ and of those who initiate treatment, nearly half drop out before completion ^16^. This suggests that there are significant barriers to receiving psychotherapy. Indeed, 75% of depressed primary care patients identify one or more practical barriers that prevent them from accessing psychotherapy ^17^.

We are developing an intervention that utilizes multiple telecommunications technologies to overcome these barriers and to insert care for depression into the patient’s life. The intervention, referred to as Technology Assisted Behavioral Intervention (TABI) uses an interactive website, e-mail, and telephone.

**2. PRIMARY AIMS AND HYPOTHESES**

The primary aim of this study is to pilot TABI for feasibility, patient acceptance, preliminary outcomes, and to examine dosing of telephone communications.

**3. OBJECTIVES AND PURPOSE**

To reduce barriers to mental health care, numerous researchers ^18-20^, as well as the 2003 President’s New Freedom Commission on Mental Health ^21^, have called for evidence-based treatments for depression that can be delivered using telecommunications. The purpose of this study is to pilot a novel intervention that can overcome numerous barriers to treatment for depression, and reduce costs.

**4. RESEARCH METHODS**

**A. Study Design**

This is a randomized, controlled pilot trial comparing an interactive website for depression management skills training with coach provided email and telephone support to a self directed version of the program and a wait list control. Patients with depression will receive up to 16 weeks of TABI. Assessments for treatment outcome will occur at baseline, week 6, week 12 and end of treatment.

**Assessments:** Assessments will consist of both web based self-report and telephone interviews. Internet administered surveys will use Surveymonkey.com, a company that ensures the security of data transmitted over the web. Self-report measures will be administered by telephone. All interviews will be audiotaped to monitor the reliability of the assessment administration.

*Mini-International Neuropsychiatric Interview (MINI)* ^22^ will be used to evaluate MDD diagnosis. Telephone administration of diagnostic interview has been well validated ^23-25^

*Hamilton Rating Scale for Depression* (HRSD) ^26^ will be used to evaluate more objective, evaluator-rated symptom severity. We will use the Medical Outcomes Study (MOS) version of the HRSD ^27^ which has been slightly altered for telephone administration by verbally assessing the final two items (ratings of psychomotor agitation and retardation based on visual observation).

*PHQ-9* ^28^ will be used to evaluate self-reported depression.

*Telephone Interview for Cognitive Status (TICS)* ^29-31^ is the most widely used telephone screening measure for cognitive status, and has been shown to be effective a screening for dementia from both stroke and Alzheimer’s.

For additional measures, see attached list.

**Treatments:** TABI will consist of a website that contains cognitive behavioral skills training tools. The TABI website production is currently being completed and will be hosted on a secure Northwestern University server or a secure Amazon 3S cloud server which provides SSL security protection with multiple passwords necessary for authentication (provides the same level of security as that of NMFF). There are two treatment groups. The first treatment group is a self-directed group. The self-directed group will be given access to the interactive website and allowed to complete the program over the course of 16 weeks. The coach assisted group will received access to the same site but will also be able to speak with a coach over the telephone and via email. Participants will speak with their coach for planned sessions of 5-15 minutes per week,. The initial session will be about 30 - 45 minutes long in order to facilitate engagement with the coach and the program, with subsequent sessions of approximately 5-15 minutes per week. If participant demonstrates improvement after the first 6 weeks, frequency of coaching session can be decreased to a level decided by coach and participant. Coaching sessions will follow a motivational interviewing based format focused on increasing participants’ motivation to continue logging in to the website. Communication between patients and coaches will also occur via e-mail. Participants in the coach assisted group will work with their coaches for the first 12 weeks of participation and then continue to have access to the site without coach assistance for an additional 4 weeks.

Participants assigned to the wait list condition will be allowed to choose which version of the program to use after 6 weeks. After 3 weeks, wait listed participants will receive a brief telephone call from study staff for a safety and wellness check. At the end of the 6 week wait list period, participants will be contacted again to select their treatment group. Participants in all three conditions will be allowed to pursue mental health care outside of the trial if desired. Participation will not limit their access to care.

**Subject Selection and Withdrawal**

**Inclusion Criteria:** 1) QIDS score of 11 or greater 2) Has a DSM-IV diagnosis of Major Depressive Episode assessed using the MINI 3) Has a telephone, e-mail account, computer, and broadband access to the Internet. 4) Is able to speak and read English. 5) Is at least 19 years of age. 6) Is able to give informed consent.

# Exclusion Criteria: 1) Has hearing or voice impairment that would prevent participation in psychotherapy. 2) Has visual impairment that would prevent use of the workbook and completion of assessment materials. 3) Meets criteria for dementia by scoring < 25 on the Telephone Interview for Cognitive Status (TICS). 4) Is diagnosed with a psychotic disorder, bipolar disorder, dissociative disorder, current post-traumatic stress disorder (PTSD), current substance abuse, or other diagnosis for which participation in a clinical trial of psychotherapy may be either inappropriate or dangerous. Patients with substance abuse diagnoses who have been clean and sober for 3 months will be admitted if otherwise eligible. 5) Is currently receiving individual psychotherapy or planning to receive psychotherapy during the 12-week treatment phase of the study. 6) Is planning to be out of town or unavailable for treatment for 4 weeks or more during the scheduled treatment time. 7) Exhibits severe suicidality, including ideation, plan, and intent. 8) Has initiated treatment with an antidepressant in past 10 days. Once patients have been on a stable dose for 10 days and do not have an appointment with a physician or psychiatrist to change this dose, the patient will be eligible based on this criterion.

**Withdrawal Criteria:** There are no specific criteria for removal of a patient from the study. A patient will be removed if continued participation is determined to constitute a danger to the patient’s health or well-being by the PI based on input from the therapists and/or evaluators. Clinical evaluators and study clinicians are required to inform the PI and study coordinator if they suspect deterioration or adverse effects in the patient. The PI and study coordinators will perform all necessary evaluations. If the PI determines the patient must be removed from the study immediately, the patient will be removed and all appropriate referrals will be made. The IRB will be informed.

**2. Number of Participants**

We will be recruiting 120 participants. 40 will be randomized to the coach assisted version of the website. 40 will be randomized to a self-guided version of the website and the final 40 will be randomized to a wait list control.

**D. Subject Recruitment**

**1. Sources**

Patients will be recruited through NMFF, NMPG, and REACH network PCP referral as well as through EPIC. NMFF and NMPG PCP referral, who have consented to be contacted about additional studies will be given information about this trial. They can contact the study coordinator if they are interested. Study information will also be posted on the Department of Preventive Medicine website. All recruitment materials will use the project name “Technology Assisted Behavioral Intervention (TABI).” All invited participants will be adults and English speaking.

**2. Initial Contact Method**

Patients at NMFF, NMPG and REACH clinics will be given flyers with contact information for this study. Additionally, PCPs will be provided a list of patient names from EPIC and they will be instructed to identify the names of patients whom we should not contact. Potential participants will be contacted via mail and invited to participate, after study staff have received approval to contact the patient from their PCP. Potential participants will not receive more than one letter per year about the trial. In order to avoid an individual receiving multiple letters, the list will be reviewed for duplicate names first by the primary care clinic computer analyst and re-examined by TABI study staff. Individuals may also request not to receive future recruitment letters.

Interested patients receive a brief telephone screen to determine whether there is any reasonable expectation that they might be eligible. If they are clearly not eligible they will be informed of this and thanked. If they are eligible they will initiate the consent process. After completing the consent process, participants will be given the option of allowing study staff to inform their primary care doctor of their participation in the study. No study data will be shared with the doctor, only a notice that they have been enrolled in the study. Whether they choose to inform their physician does not affect their ability to participate in the study or the treatment they receive.

**B. Data Analysis**

Data will be analyzed by our biostatistician. Since this is a feasibility study, we will be exploring the data via descriptive statistics and effect size estimation.

**E. Consent Process and Documentation**

When an individual calls in response to a flier, he or she will be given a brief screen to determine whether they are at all eligible for the study. Individuals will be told detailed information about the study, including elements found in both the verbal and paper consents. A date and time for the initial interview will be scheduled during this first phone call as well.

Following the phone call, the participant will be emailed the digital version of the consent form. Subjects agree to participate by checking a yes box and typing in their name. They are instructed to print out the consent form for their records and email the consent back to study staff. The day before their appointment, they will be sent an email with a link to the self-report measures, asking them to fill these out as close to the interview as possible.

At the previously scheduled date and time, the participant will be consented verbally. Because there will be no physical contact between study staff and study participants, the verbal consent is critical to ensuring that patients understand the study procedures and what specifically they are consenting to. A copy of the verbal consent is attached. The staff member conducting the consent must read each section, ensure that the prospective participant understands that section, initial that section, and then proceed to the next section. If, at the end of this verbal process, the patient understands the study and gives verbal agreement, and has returned a completed consent form via email, the interview will proceed. Patients may decide at any time in the process to decline to participate. If a consent form is returned via email, consent will be implied. If data is returned without a completed consent, a research assistant will contact the participant and discuss the consent process. If we are unable to obtain consent, the data will be destroyed and will not be included in subsequent analyses.

**F. Procedures**

**1. Study Procedures**

**1.1 Initial Interview:** The initial interview will take place when the participant has been verbally consented and has indicated that s/he has read and signed the consent form.. The initial interview will last approximately 30-45 minutes (including verbal consent) and will be audiotaped to ensure reliability in assessment. The participant will complete the self-report questionnaires on-line as close to the interview as possible. The questionnaires will take approximately 45-60 minutes to complete. A list of these measures is included in the Appendix. Based upon these results a determination will be made as to the patient’s eligibility. The patient will be informed of his/her eligibility within 72 hours of his/her assessment. If the patient is eligible, the patient will be randomized upon receipt of the written consent document. Under no circumstances will any patient initiate treatment prior to receipt of the consent document.

**1.2 Assessments:** Follow-up assessments will occur at weeks 6, 12 and 16 (end of treatment). All interview assessments will be conducted over the telephone. Self-report questionnaires will be completed by Internet administration. Participants will also be asked about their impressions of the website and the individual modules. Participants who feel strongly, either positively or negatively, about the site will be invited to come in to the Behavioral Medicine Research Laboratory in the Department of Preventive Medicine in order to provide in person feedback about the current website and proposed changes to its design and content. Since this is a pilot study, it is important to ascertain both the positives and negatives about the site so that it can be modified to be more effective in the future. If a participant is not interested in participating in the in-person interview, it will not affect his or her ability to participate in the rest of the study.

The primary danger to the patient with depression is self harm. No study procedures are known to aggravate that risk, but we have nevertheless developed procedures to ensure safety of participants. Suicidality is assessed both by interview using the MINI and the QIDS, and by self-report using the PHQ-9. If the PHQ-9 suicide item is a 3, an automated computer program will alert the clinicians on staff to contact the patient and assess their safety. As our clinicians do not provide after-hours emergency support, the safety assessment would be performed the next business day if the alert is received after hours. However, the website will immediately refer patients scoring a 3 on the PHQ-9 suicide items to 911 or 1-800-SUICIDE for emergency services. If the patient reports suicidal ideation and intent upon interview, the study coordinator and PI will be notified immediately. In either case, the patient will be contacted and evaluated, and all necessary steps will be taken to ensure the patient’s safety, up to and including calling emergency services to go out to the patient’s location, perform a safety check and hospitalize the patient if necessary.

**2. Time**

The total amount of time required by patients is approximately 45 minutes; this includes time to review the materials provided to them, sign consent document, and complete the self-report questionnaires. The in-person interview will last up to 2.5 hours.

**3. Study Sites**

This study will be conducted through the Department of Preventive Medicine at the Feinberg School of Medicine.

**G. Risks/Discomforts**

The risks from completing the self-report measures and interviews are minimal, but it is possible that the participant may experience emotional discomfort or increased anxiety as a result of answering questions about their mental health. The participant will be reminded that s/he has the option of stopping if s/he finds completing the self-report measures to be overly distressing. There are no known risks associated with TABI, as this is the first feasibility study. Patients will be permitted to continue antidepressant medications. As such, participation in this study is not expected to pose any significant risk to participants.

**H. Treatment and Compensation for Injury**

Participant risks in this study are minimal. The only risk is emotional discomfort. The participant will be reminded that s/he has the option of stopping if s/he finds completing the self-report measures to be overly distressing.

**I. Alternatives**

The alternative to participating in this study is not participating.

**J. Costs to the Subject**

There is no cost to the patient for participating in the study.

**K. Reimbursement of Subjects**

Subjects will be sent a $20.00 check after each of the first three assessments (baseline, 6 week and 12 week). Participants will receive $40.00 for completion of the final assessment at Week 16. Participants in the wait list control condition will be assessed at same four time points as other participants, but will also receive an assessment at Week 6 and Week 12 of receiving the intervention. They will receive a check for $20 for the Week 6 assessment and a $40 check for the Week 12 assessment. If selected, participants will receive an additional $40 for participating in the in-person interview.

**L. Confidentiality of Records**

No information that identifies participation will be released without prior consent except as specifically required by law. Resulting data will be kept in a locked, password protected database in which only the PI and research team have access.

**5. QUALIFICATIONS OF INVESTIGATORS**

Principal Investigator: David C. Mohr, Ph.D. is Professor in the Department of Preventive Medicine at the Feinberg School of Medicine.

**6. BIBLIOGRAPHY**

1. Kessler RC, McGonagle KA, Zhao S et al. Lifetime and 12-month prevalence of DSM-III-R psychiatric disorders in the United States. Results from the National Comorbidity Survey. *Arch Gen Psychiatry* 1994;51:8-19.

2. Kessler RC, Berglund P, Demler O et al. The Epidemiology of Major Depressive Disorder: Results From the National Comorbidity Survey Replication (NCS-R). *Jama* 2003;289:3095-105.

3. Wells K, Miranda J, Bauer M et al. Overcoming barriers to reducing the burden of affective disorders. *Biol Psychiatry* 2002;52:655.

4. Whooley MA, Simon GE. Managing depression in medical outpatients. *N Engl J Med* 2000;343:1942-50.

5. Whooley MA, Stone B, Soghikian K. Randomized trial of case-finding for depression in elderly primary care patients. *J Gen Intern Med* 2000;15:293-300.

6. Hollon S, Munoz R, Barlow D et al. Psychosocial intervention development for the prevention and treatment of depression: promoting innovation and increasing access. *Biol Psychiatry* 2002;52:610.

7. Elkin I, Shea MT, Watkins JT et al. National institute of mental health treatment of depression collaborative research program: General effectiveness of treatments. *Archives of General Psychiatry* 1989;46:802-808.

8. Elkin I. The NIMH treatment of depression collaborative research program: Where we began and where we are. In: Bergin AE, Garfield SL, eds. *Handbook of psychothrapy and behavior change, 4th edition*. New York: Wiley & Sons; 1994.

9. Robinson LA, Berman JS, Neimeyer RA. Psychotherapy for the treatment of depression: a comprehensive review of controlled outcome research. *Psychol Bull* 1990;108:30-49.

10. Brody DS, Khaliq AA, Thompson TL, 2nd. Patients' perspectives on the management of emotional distress in primary care settings. *J Gen Intern Med* 1997;12:403-6.

11. Dwight-Johnson M, Sherbourne CD, Liao D, Wells KB. Treatment preferences among depressed primary care patients. *J Gen Intern Med* 2000;15:527-34.

12. Bedi N, Chilvers C, Churchill R et al. Assessing effectiveness of treatment of depression in primary care. Partially randomised preference trial. *Br J Psychiatry* 2000;177:312-8.

13. Priest RG, Vize C, Roberts A, Roberts M, Tylee A. Lay people's attitudes to treatment of depression: results of opinion poll for Defeat Depression Campaign just before its launch. *Bmj* 1996;313:858-9.

14. Churchill R, Khaira M, Gretton V et al. Treating depression in general practice: factors affecting patients' treatment preferences. *Br J Gen Pract* 2000;50:905-6.

15. Weddington WW, Jr. Adherence by medical-surgical inpatients to recommendations for outpatient psychiatric treatment. *Psychother Psychosom* 1983;39:225-35.

16. Wierzbicki M, Pekarik G. A meta-analysis of psychotherapy dropout. *Professional Psychology: Research and Practice* 1993;24:190-195.

17. Mohr DC, Hart SL, Howard I et al. Barriers to psychotherapy among depressed and nondepressed primary care patients. *Ann Behav Med* 2006;32:254-8.

18. Tanvetyanon T. Telephone psychotherapy and care management for depression. *Jama* 2004;292:2720; author reply 2720-1.

19. Maheu MM, Pulier ML, Wilhelm FH, McMenamin JP, Brown-Connolly NE. The mental health professional and the new technologies: A handbook for practice today. Mahwah, NJ: Lawrence Erlbaum Associates; 2005.

20. Mohr DC, Hart SL, Julian L et al. Telephone-administered psychotherapy for depression. *Arch Gen Psychiatry* 2005;62:1007-14.

21. President's New Freedom Commission on Mental Health. Achieving the promise: Transforming mental health care in America. Rockville, MD: Department of Health and Human Services; 2003.

22. Sheehan DV, Lecrubier Y, Sheehan KH et al. The Mini-International Neuropsychiatric Interview (M.I.N.I.): the development and validation of a structured diagnostic psychiatric interview for DSM-IV and ICD-10. *J Clin Psychiatry* 1998;59 Suppl 20:22-33;quiz 34-57.

23. Rohde P, Lewinsohn PM, Seeley JR. Comparability of telephone and face-to-face interviews in assessing axis I and II disorders. *Am J Psychiatry* 1997;154:1593-8.

24. Simon GE, Revicki D, VonKorff M. Telephone assessment of depression severity. *J Psychiatr Res* 1993;27:247-52.

25. Ruskin PE, Reed S, Kumar R et al. Reliability and acceptability of psychiatric diagnosis via telecommunication and audiovisual technology. *Psychiatr Serv* 1998;49:1086-8.

26. Hamilton M. A rating scale for depression. *Journal of Neurology, Neurosurgery, and Psychiatry* 1960;23:56-62.

27. Potts MK, Daniels M, Burnam MA, Wells KB. A structured interview version of the Hamilton Depression Rating Scale: evidence of reliability and versatility of administration. *Journal of Psychiatric Research* 1990;24:335-350.

28. Kroenke K, Spitzer RL, Williams JB. The PHQ-9: validity of a brief depression severity measure. *J Gen Intern Med* 2001;16:606-13.

29. Desmond DW, Tatemichi TK, Hanzawa L. The telephone interview for cognitive status (TICS): reliability and validity in a stroke sample. *International Journal of Geriatric Psychiatry* 1994;9:803-807.

30. Brandt J, Spencer M, Folstein M. The Telephone Inteview for Cognitive Status. *Neuropsychiatr Neuropsychol Behav Neurol* 1988;1:111-117.

31. Welsh KA, Breitner JCS, Magruder-Habib KM. Detection of dementia in elderly using telephone screening of cognitive status. *Neuropsychiatry, Neuropsychology, and Behavioral Neurology* 1993;6:103-110.

32. Belloc NB, Breslow L, Hochstim JR. Measurement of physical health in a general population survey. *Am J Epidemiol* 1971;93:328-36.

32. Wright, JH. Wright, AS., Salmon, P et al. Development and initial testing of a multimedia program for computer-assisted cognitive therapy. Am J Psychother 2002;56: 76-86.
